# PANDEMIC HARDSHIP AMONG SENIOR LIVING RESIDENTS: WHO WAS MOST VULNERABLE?

**DOI:** 10.1093/geroni/igae098.3680

**Published:** 2024-12-31

**Authors:** Mollie George, Lindsay Wilkinson, Julie Masters, Julie Blaskewicz Boron

**Affiliations:** University of Nebraska Omaha, Lincoln, Nebraska, United States; University of Nebraska Omaha, Omaha, Nebraska, United States; University of Nebraska Omaha, Omaha, Nebraska, United States; University of Nebraska Omaha, Omaha, Nebraska, United States

## Abstract

The COVID-19 pandemic presented unprecedented challenges for older adults. While there has been an abundance of research on the pandemic-related experiences of community-dwelling older adults, little research has examined those in senior living communities (SLCs). This is significant given the restrictions enforced in SLCs to reduce the risk of infection and potential hospitalization or death. The current research aimed to investigate the pandemic-related experiences and correlates of pandemic hardship among those residing in SLCs. Survey data were collected during the pandemic from adults age 60 or older living in a group of SLCs in Nebraska. Most respondents in the analytic sample (n=726; Mage=84, SD=7, range: 60-100; 73% female) resided in independent living (86%). Drawing on descriptive statistics and linear regression analysis, the results revealed a moderate to high amount of pandemic hardship (M=4, scale: 1-6). The most prevalent negative pandemic-related experiences included difficulty visiting family and friends (91% of respondents agreed), missing out on important events (83%), and perceiving little control over their lives (68%). In models accounting for sociodemographic and health characteristics, residents most vulnerable to the hardship of the pandemic were less likely to be never married (ps< 0.05), had lower perceived health (p< 0.05), and scored lower on resilience (p< 0.001). This research reveals the pandemic-related experiences of an understudied population of independent and assisted living residents, including areas of their lives they perceived to be most impacted by social distancing. This can be helpful in identifying vulnerable groups in need of support during times of crisis.

